# Effect of insulin–glucose metabolism compared with obesity on adipose *omentin* gene expression in different models of diabetic C57BL/6 mice

**DOI:** 10.1186/s13098-019-0460-8

**Published:** 2019-08-14

**Authors:** Golnaz Goodarzi, Amirreza Shirgir, Sadegh Alavi, Amirhosein Khoshi

**Affiliations:** 1 Department of Clinical Biochemistry, School of Medicine, North Khorasan University of Medical Sciences, Bojnurd, Iran; 2 Student Research Committee, School of Medicine, North Khorasan University of Medical Sciences, Bojnurd, Iran

**Keywords:** Omentin, Diabetes mellitus, Obesity, Insulin, C57BL/6 mice

## Abstract

**Background:**

Omentin, releasing by adipose-tissue may be related to glucose metabolism. The omentin circulating levels and the related mRNA expression in visceral adipose-tissue are different in types of diabetes and the exact function of this molecule is still unknown. The aim of this study was to examine *omentin* gene expression in adipose-tissues of type-1 and type-2 diabetic mice for the investigation of the effects of fat-mass and insulin–glucose metabolism.

**Methods:**

In this study, 36 C57BL/6 mice were divided into four experimental groups, including control, type-1 diabetes (inducted by streptozotocin), type-2 diabetes with obesity (high-fat diet + low-dose-streptozotocin [HFD + STZ]), and type-2 with normal weight (normal-pellet diet + low-dose-streptozotocin [NPD + STZ]). The present study involved the measurements of oral-glucose-tolerance-test and the levels of biochemical parameters, including blood glucose, omentin, insulin, lipid-profile, as well as aminotransferases. In addition, the *omentin* mRNA expression was evaluated by real-time polymerase-chain-reaction.

**Results:**

The results of *omentin* gene expression analysis showed a significant difference between mRNA expressions in the experimental groups. The plasma omentin levels were significantly higher in type-1 diabetes group and lower in type-2 diabetes with NPD + STZ; however, the plasma omentin levels were not changed in the HFD + STZ group. In addition, the findings of serum-biochemical analysis revealed significant differences, compared to the control-group.

**Conclusions:**

The *omentin* expression may be affected by insulin and glucose levels in different types of diabetes more than fat-mass, and due to the local activity, the serum omentin may not comply with its gene expression.
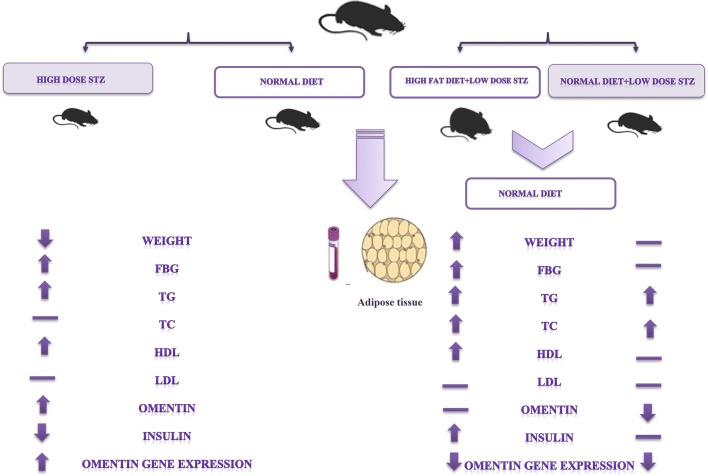

## Background

The prevalence rate of diabetes paralleling the overweight and obesity is growing up around the world at an alarming rate [1, 2]. Increasing morbidity and mortality following diabetes has turned it into one of the most important health and economic issues [2, 3]. The main cause of type 1 diabetes (T1D) is autoimmune-mediated destruction of beta cells in the pancreatic islet [4, 5]. Approximately, there are 95% of diabetic patients with type 2 diabetes (T2D) that mainly illustrated by hyperglycemia due to the defects in insulin secretion, insulin action, or both of them [4, 6].

Obesity, especially abdominal or visceral obesity is one of the major risk factors for metabolic disorders, such as insulin resistance, T2D, dyslipidemia, and cardiovascular disease [7]. In fact, the relation between obesity and T2D is referred to the activity and function of adipose tissues [8]. Multiple studies demonstrated adipose tissue secretes many biologically active substances as known adipokines, such as leptin [9], adiponectin [10], and omentin [11].

Omentin, including two isoforms, was identified as a novel secretory protein with 313 amino acids that is mainly expressed in visceral adipose tissue rather than subcutaneous adipose tissues in human [11, 12]. Furthermore, omentin is mainly expressed in intestinal Paneth cells of mice [13]. The *omentin* gene is located in the 1q22-q23 chromosomal region, which has been associated with T2D in different populations [14–17]. Omentin-1 was shown to be a main circulating isoform in human plasma [3]. The exact role of omentin, as a new biological substance, is not well-described in the literature. However, the results of an in vitro study demonstrated that omentin can increase insulin-mediated glucose uptake by activating the protein kinase Akt or protein kinase B [12]. According to the results of recent studies, it was shown that plasma levels of omentin are different in T1D and T2D [5, 6]. Moreover, based on the evidence it was revealed that underweight subjects had higher plasma omentin-1 levels, compared to obese and overweight cases [3, 18]. Furthermore, serum omentin level and its gene expression in adipose tissue have demonstrated a negative correlation with overweight/obesity and insulin resistance [3, 19]. So far, the investigation of insulin–glucose metabolism variations in diabetes with and or without obesity on *omentin* expression in adipose tissue has not been performed, while some studies have mentioned that mice adipose tissue may not have an important role in the secretion of omentin [12].

Therefore, due to the probable potential role of omentin as an insulin sensitizer, the predominant expression of *omentin* in adipose tissue and its presence in circulation, it was decided to determine the serum omentin levels and the related gene expression in animal models as normal subjects, T1D, T2D with normal weight as well as obesity, and analyze the relationship between *omentin* gene expression levels with plasma glucose, insulin, omentin, and other biochemical parameters.

## Materials and methods

### Animals study

This study was conducted on a total of 36 male C57BL/6 mice (Pasteur Institute, Iran) with 8 weeks of age and approximately 20–25 g. All procedures were approved by the Ethics Committee of North Khorasan University of Medical Sciences (ethical code: IR.nkums. REC.1396.24). The animals were kept in a clean cage under controlled condition (25 ± 2 °C) and humidity (50%) with a 12/12 h light/dark cycle. All the mice were fed with a normal pellet diet (NPD) and free water 1 week before the initiation of the experiment and allowed to acclimatize to the laboratory environment. All the mice were divided into four groups with twelve animals in control group and eight animals for each experimental groups as given below: group (1) healthy mice as controls fed with normal chow including six animals as control for T1D mice, and six animals as control for T2D mice, group (2) the mice with T1D induced by high doses of streptozotocin (STZ), group (3) the mice with T2D induced by high-fat diet + STZ (HFD + STZ), and group (4) the mice with T2D induced by NPD + STZ (NPD + STZ).

### Type 1 diabetes induction

Type 1 diabetes was induced in anesthetized and overnight fasted mice of group 2 by a single intraperitoneal injection of STZ (65 mg/kg) in a 2% (w/v) solution of 0.1 M citrate buffer (pH 4.5), while the control group received exclusively citrate buffer [20]. After 1 h, the animals were fed with standard food and water. After 72 h of injection, the blood glucose levels were estimated and monitored every week during the experiment until 9 weeks using the Accu-Chek glucose meter. The STZ-treated mice with blood glucose levels more than 11.1 mmol/L were considered as diabetic and used for the present study.

### Type 2 diabetes induction

The mice were divided into two dietary groups, namely HFD + STZ and NPD + STZ. After 7 weeks of dietary manipulation, intraperitoneal with a low dose of STZ (45 mg/kg) was injected into the mice from each dietary group [21]. The food intake, body weight, and fasting blood glucose were measured every week until 12 weeks. The inclusion criterion was the mice with a fasting blood glucose level higher than 8.3 mmol/L 4 weeks after the injection.

### Glucose tolerance test

Four weeks after STZ injection, an oral glucose tolerance test was performed following 14-h fasting in groups 3 and 4. Plasma glucose concentrations were measured in blood samples that were taken from the tail using Accu-Chek glucose meter at 0, 15, 30, 60 and 120 min post administration of glucose (3 g/kg) [22].

### Biochemical measurements

At the end of the experiment, fasting blood specimen was collected from each group to determine glucose, insulin, omentin, and biochemical parameters, including lipid profile, aspartate aminotransferase (AST) and alanine aminotransferase (ALT). The plasma samples were kept at − 80 °C until the assay. Fasting blood glucose, total cholesterol, triglyceride, and high-density lipoprotein cholesterol (HDL-C) were measured using enzymatic methods (PishtazTeb, Iran). The Friedewald equation was used to calculate low-density lipoprotein cholesterol. Plasma insulin (Abnova, Taiwan) and omentin (MyBioSource, USA) levels were measured by the enzyme-linked immunosorbent assay (ELISA) following the manufacturer’s protocols, respectively.

### RNA extraction and real-time quantitative polymerase chain reaction

Total RNA was isolated from the frozen adipose tissues by column method (Bio Basic, Canada) and according to the manufacturer’s instruction RNA quality was determined by measuring the 260/280 nm ratio. Moreover, the concentration was determined by an ultraviolet spectrophotometer (PCR^max^Lambada, Japan). Complementary DNA synthesis was performed with 500 ng RNA from each sample using PrimeScript Reverse Transcript Reagent Kit (TaKaRa Inc., Japan). Quantitative real-time polymerase chain reaction (qRT-PCR) analysis was carried out to quantify the expression of *omentin* (as a target gene), compared with *Beta 2 Microglobulin (B2M)* (as a reference gene) using SYBR Premix Ex Taq П (TaKaRa Inc., Japan) by Rotor-Gene 6000 qPCR machine (Qiagen, Germany). The specific primers for *omentin* and *B2M* were F-5′-GCTGAAGAGAACCTGGAC-3′ and R-5′-AATAGAGACCATCTTGTGC-3′, F-5′-CTTCAGCAAGGACTGGTC-3′ and F-5′-TCTCGATCCCAGTAGACG-3′, respectively. The stages of tree steps qPCR were pre-denaturation at 95 °C for 5 min, frothy cycles at 95 °C for 10 s, 55 °C (for *omentin*) and 57 °C (for *B2M*) for 20 s, as well as 62 °C for 30 s. Melting curve analysis was performed by increasing the temperature (1 °C) from 52 to 95 °C with continues fluorescence acquisition. Relative expression in *omentin* mRNA levels was calculated using the 2^−ΔΔCT^ method and normalized based on *B2M* mRNA levels.

### Statistical analysis

All results are presented as mean ± SD and were analyzed using the SPSS 18. Differences between groups were calculated using either Student’s t test or one way ANOVA (Tukey’s test) analysis. The association between variables was calculated by using Pearson’s for parametric variable and Spearman Rho correlation test for non-parametric. P values < 0.05 were considered statistically significant.

## Results

### Biochemical parameters and body mass

**Fig. 1:**
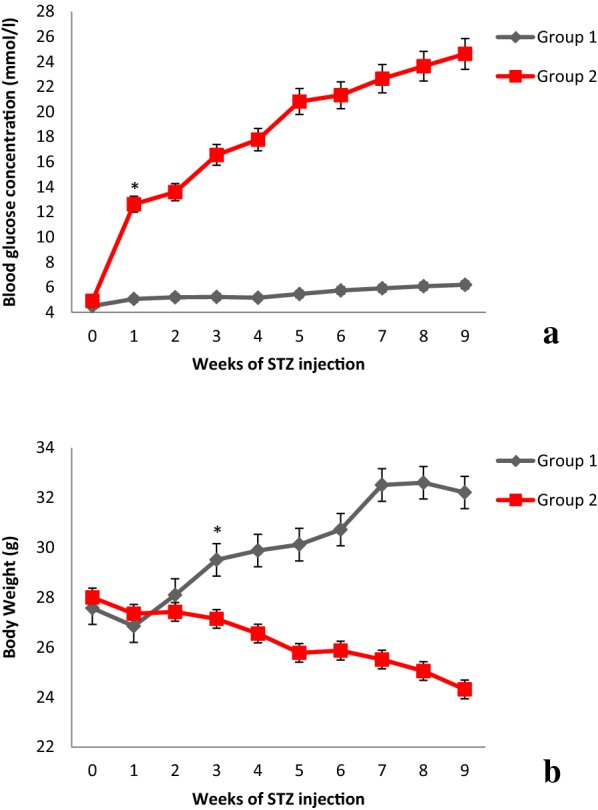
Blood glucose concentration (**a**) and body weight (**b**) during 9 weeks after STZ injection in type 1 diabetic group compared to control group

The results showed no significant differences in level of body weight, FBG and other biochemical parameters at beginning of study. While 72 h after STZ injection, mice treated with STZ (group 2) showed significant hyperglycemia comparing to control group (12.31 ± 1.85 vs. 5.16 ± 0.48 mmol/L) (P = 0.001) (Fig. 1a). Three weeks after beginning of diabetes, body weight in type 1 diabetic group was significantly decreased in compare to control group and continue till end of experiment (24.31 ± 0.5 g, vs. 32.21 ± 1.7 g), respectively (P = 0.001) (Fig. 1b). In addition water and food intake increased, but data not shown.

**Fig. 2:**
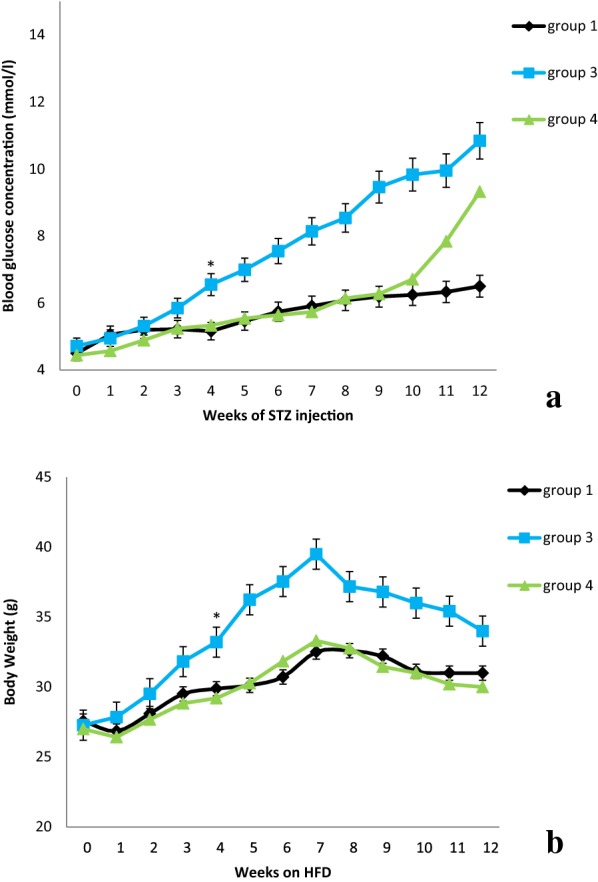
Blood glucose concentration (**a**) and body weight (**b**) during 12 weeks after HFD and STZ injection on 7th week in type 2 diabetic groups compared to control

Feeding the mice with high fat diet for 12 weeks, showed significantly increases in FBG and body weight in group 3 in compare to group 4. However, injection of STZ (45 mg/kg) in 7th week induced hyperglycemia and decreased body weight of HFD + STZ and NPD + STZ, respectively, while body weight and FBG in HFD + STZ group were still significantly higher than NPD + STZ (Fig. 2).

Changes in levels of other biochemical parameters, as well as plasma omentin and insulin levels were evaluated in all groups at the end of experiment and illustrated in Table 1.

**Table 1:** Biochemical parameters in serum

Parameters^b^	Study groups^a^						
	Control (Group 1)	T1D (Group 2)	P value	HFD + STZ (Group 3)	P value	NPD + STZ (Group 4)	P value
FBG	6.19 ± 0.6	21.99 ± 6.62	*0.000*	9.46 ± 0.88	*0.000*	6.27 ± 0.52	*0.805*
TG	1.30 ± 0.09	1.67 ± 0.12	*0.025*	2.16 ± 0.11	*0.000*	1.81 ± 0.29	*0.038*
TC	3.51 ± 0.27	3.99 ± 0.38	*0.055*	7.75 ± 0.67	*0.000*	4.28 ± 0.70	*0.086*
LDL	1.45 ± 0.21	1.48 ± 0.14	*0.815*	1.61 ± 0.12	*0.155*	1.57 ± 0.21	*0.333*
HDL	1.16 ± 0.14	1.42 ± 0.11	*0.006*	2.67 ± 0.65	*0.001*	1.69 ± 0.29	*0.097*
AST	170 ± 22.86	343.33 ± 31.41	*0.001*	137 ± 19.10	*0.063*	125.42 ± 26.12	*0.237*
ALT	13 ± 3.22	185 ± 27.92	*0.0001*	26.85 ± 4.98	*0.0001*	26 ± 8.83	*0.037*
Omentin	3.89 ± 0.487	5.65 ± 0.55	*0.01*	3.26 ± 0.41	*0.176*	2.01 ± 0.52	*0.001*
Insulin	2.38 ± 0.65	0.66 ± 0.18	*0.022*	4.68 ± 0.81	*0.038*	1.75 ± 0.58	*0.159*

Significant values are in italics

Values are mean ± SD. Serum parameters are measured on 9th week for group 2, and 12th week for groups 3 and 4

^a^Control group: the regular chow (Group 1); T1D: type 1 diabetes (Group 2), HFD + STZ: high fat diet and low dose of STZ (Group 3), NPD + STZ: normal pellet diet and low dose STZ (Group 4)

^b^FBG (mmol/L); TG (mmol/L), TC (mmol/L), LDL density lipoprotein (mmol/L), HDL (mmol/L), AST (IU/L), ALT (IU/L), omentin (ng/mL), insulin (μIU/mL)

During the present study, the increase of blood glucose, water consumption, and urination indicated the successful induction of T1D. The T2D mice which were fed with HFD increased the body weight, triglyceride, and cholesterol circulating concentrations, compared to the control group. In addition, plasma glucose concentration and insulin level were increased in the mice with HFD, which together show impaired glucose tolerance and insulin resistance.

As results showed fasting blood glucose elevated following diabetes induction. The serum triglycerides were increased in T1D, HFD + STZ and NPD + STZ type 2 diabetic mice, significantly. The serum total cholesterol also increased in HFD + STZ mice, significantly. LDL-C levels were not change in different groups, whereas the HDL-C levels were elevated significantly in T1D and HFD + STZ type 2 diabetic mice. The serum omentin levels were increased in T1D (P = 0.01) whereas omentin levels were reduced in NPD + STZ mice (P = 0.001), but not in HFD + STZ, in compare to control group. Moreover, serum aminotransferases ALT was increased in T1D (14-fold) and T2D (twofold) diabetic mice. Moreover, AST values significantly increased in T1D group (twofold), but not in the other study groups.

The blood glucose levels after an oral glucose tolerance test in T2D mice and its control groups have mentioned in Table 2. Since the blood glucose concentrations were similar in both groups, therefore OGTT were carried out to compare insulin resistance. The results have shown the rate of glucose disappearance in NPD-fed was significantly higher than HFD-fed.

**Table 2:** Oral glucose tolerance test in two models of type 2 diabetes

T2D models^a^	HFD + STZ	NPD + STZ	P value
Time			
Time 0	10.84 ± 1.60	9.54 ± 1.88	0.190
Time 15	26.76 ± 3.51	22.10 ± 1.30	0.013
Time 30	24.93 ± 3.10	22.80 ± 2.97	0.30
Time 60	26.75 ± 3.30	18.70 ± 4.09	0.036
Time 120	22.02 ± 4.86	10.56 ± 2.11	0.001

Values are mean ± SD

^a^Oral glucose tolerance test in type 2 diabetes conditions include high-fat diet + STZ and normal pellet diet + STZ

### Omentin gene expression

**Fig. 3:**
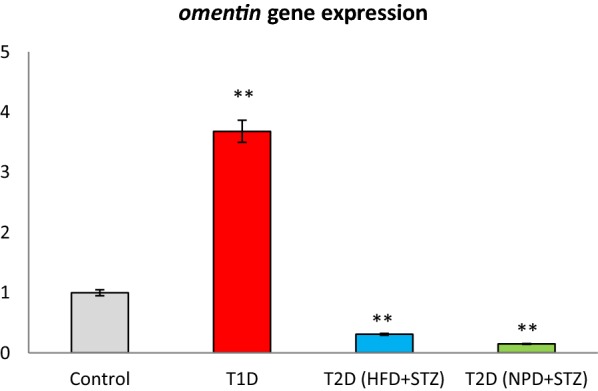
Adipose tissue related *omentin* gene expression in type 1 and type 2 diabetic mice models. Values are mean ± SD. Control group: the regular chow (Group 1); T1D: type 1 diabetes (Group 2), T2D (HFD + STZ): high fat diet with low dose STZ (Group 3), T2D (NPD + STZ): normal pellet diet with low dose STZ (Group 4)

We examined the changes on the gene expression of *omentin* in T1D and T2D models include HFD + STZ and NPD + STZ states. As results showed the *omentin* gene expressions decreased in T2D mice which induced by HFD and NPD feeding with low doses of STZ, whereas *omentin* expression elevated in following T1D (P < 0.0001) (Fig. 3).

## Discussion

In the present study, the *omentin* gene expression was examined in adipose tissues of T1D and T2D mice models that were induced by higher and lower doses of STZ with normal pellet and HFD diet for the investigation of the effects of fat mass on insulin metabolism in different types of diabetes. Many studies have attempted to detect the associations between serum omentin levels and different types of diabetes. However, a few studies have been carried out to find the molecular mechanism of the variation of *omentin* gene expression in different types of diabetes and its relationship with serum biochemical parameters in T1D and T2D.

The obtained results of this study revealed that the plasma insulin levels were significantly decreased in type 1 diabetic mice. In addition, there was a significant increase in insulin levels in HFD + STZ type 2 diabetic mice. However, no significant difference was observed in NPD + STZ type 2 diabetic mice, compared to the control group.

Regarding the plasma omentin levels, there was a significant difference in T1D and NPD + STZ model of T2D; nonetheless, omentin levels in T2D with HFD + STZ were not statistically significant. However, the investigation of *omentin* gene expression in adipose tissues of the studied groups showed that the *omentin* expression level was significantly higher in T1D mice; however, the *omentin* expression resulted in a significant reduction in both models of T2D mice.

Despite no significant differences in plasma omentin levels in HFD + STZ type 2 diabetes, the obtained results revealed that the omentin levels of HFD + STZ mice were lower than those of T1D. In addition, the gene expression of *omentin* in adipose tissues of HFD + STZ mice was significantly lower than those of both T1D and control groups. These results suggested that the circulating omentin concentrations may not always be associated with adipose tissue mass and the physiological/hormonal role of omentin may be local in insulin signaling.

It has been shown that the dose-dependent administration of glucose and insulin to human omental adipose tissues results in a reduction of *omentin*-*1* expression. In addition, insulin–glucose infusion in healthy individuals can significantly decrease plasma omentin-1 concentration. Therefore, the above-mentioned results revealed that omentin-1 production is under glucose and insulin regulation [23].

It has demonstrated that the underweight individuals had higher plasma omentin-1 level than those who were overweight. Furthermore, plasma omentin-1 inversely correlated with body mass index and insulin resistance; however, it was positively associated with HDL-C levels. It has also shown that the *omentin* gene expression decreased with obesity. Taken together these results clearly show that obesity reduces serum omentin-1 concentration in adults and adolescents [3].

It has reported that omentin is preferentially expressed in mouse intestinal Paneth cells [13]. However, the results of several studies have shown that *omentin* gene is mainly expressed in human visceral adipose tissue. Moreover, it has observed that *omentin* is scarcely expressed in mouse visceral adipose tissue. These findings suggest that omentin may play a more important role in adipose tissue of humans than in mice [12, 24].

In the present study, it was demonstrated that *omentin* was expressed in adipose tissues of C57BL/6 mice and its expression is changed in different types of diabetes. According to the results of the present study, it was revealed that the *omentin* mRNA levels in T1D model increased 3.68-fold and had a positive correlation with serum omentin levels. Moreover, the *omentin* mRNA levels in T2D models, including HFD + STZ and NPD + STZ, were attenuated to 0.31- and 0.15-fold, respectively. There was also no significant correlation between the related mRNA levels and serum omentin levels. However, a negative association was observed between *omentin* mRNA and serum ALT levels in HFD + STZ model.

Based on the obtained results of the present study, it was also shown that *omentin* gene expression had a positive correlation with the animal weight, while it had a negative relation with the fasting blood glucose in NPD + STZ model. These findings reflected that not only *omentin* was expressed in mice visceral adipose tissue but also it can change based on different metabolic conditions.

It has reported that the omentin concentrations of fasting and 2 h following oral glucose tolerance test were significantly decreased in diabetic subjects, as well as in subjects with IGT (impaired glucose tolerance) when compared to the control group [6]. In addition, it was observed that plasma omentin concentrations in pre-diabetic or diabetic obese patients were lower than the cases with normal fasting blood glucose in a cross-sectional study [25]. Furthermore, there was a significantly reduced serum omentin concentration in T2D patients with gender and BMI adjusted with healthy controls. Moreover, their results reflected that serum omentin level negatively correlated with BMI, fasting blood glucose, and HOMA-IR [19]. According to the findings of the aforementioned study, it was revealed that the serum omentin-1 levels are not affected only by the increase of fat mass and more omentin concentrations can be affected by insulin and glucose variations. In the current study, serum omentin concentrations in T2D mice, which were induced by NPD and lower doses of STZ, were reduced, followed by the decreased gene expression in adipose tissues. However, the serum omentin levels in T2D mice induced with HFD and a low dose of STZ did not decrease and were not compatible with its decreased gene expression in adipose tissue. Thus, there may be other sources for circulating omentin. Similar to above studies [6, 19], the findings of the present study revealed that serum omentin levels could be reduced in T2D, although it may not be observed in all the conditions of T2D.

It is reported serum omentin concentration was significantly lower in children with T1D than in control children [5]. However, in current study, serum omentin concentrations in T1D mice raised, followed by the increased gene expression in adipose tissues.

It has shown that serum omentin level is significantly reduced in obese mice, compared to the non-obese ones [18]. However, another study demonstrated that serum omentin levels were similar between obese and non-obese groups [26]. Similarly, it was no significant difference between the obese diabetic mice and non-obese normal controls in this study.

It has demonstrated that serum omentin level was increased in diabetic rats induced with STZ [27]. Similarly, both adipose tissues related to *omentin* gene expression and serum omentin levels in T1D model were increased in the current study. Since the insulin production was suppressed in T1D mice, it hypothesized that the *omentin* gene expression and its serum levels rise to compensate the insulin deficiency in type 1 diabetes.

In a study that was conducted to experimental T2D in rats, there was no change in omentin levels in the groups including HFD and low dose of STZ [28]. Similarly, the serum omentin levels in C57BL/6 T2D mice induced with HFD and a low dose of STZ did not significantly change in the present study.

The obtained results of the present study showed that the *omentin* mRNA levels may be affected by the changes of fat tissue mass in mice model. Moreover, the changes in serum omentin levels could depend on different metabolic conditions. On the other hand, the source of serum omentin may be different according to different health and disease conditions.

## Conclusions

The obtained results revealed that the mice adipose tissue had an important role in the production of omentin. Additionally, the circulating omentin may change, followed by different conditions and could be related to visceral fat mass. The adipose tissue related *omentin* gene expression and the serum omentin levels of type 1 and NPD + STZ type 2 diabetic mice model raised and reduced, respectively. However, HFD + STZ type 2 diabetic model mice did not show any significant differences in serum omentin levels. However, the adipocyte related *omentin* gene expression significantly decreased. The increased *omentin* gene expression showed a significantly negative correlation with serum insulin levels in T1D mice. Nonetheless, the decreased *omentin* gene expression positively correlated with serum insulin levels in T2D models. Consequently, it was concluded that the increase of fat mass and insulin–glucose metabolism can have important roles in the expression of *omentin*. Moreover, the serum omentin may not always comply with its gene expression and the effect of insulin–glucose variations in different types of diabetes on *omentin* gene expression may be more than increased fat mass.

## Data Availability

Data sharing not applicable to this article.

## Abbreviations

ALT: alanine aminotransferase
AST: aspartate aminotransferase
*B2M*: *Beta 2 Microglobulin*
DNA: deoxy ribonucleic acid
ELISA: enzyme-linked immunosorbent assay
FBG: fasting blood glucose
g: gram
HDL-C: high density lipoproteins-cholesterol
HFD: high fat diet
IGT: impaired glucose tolerance
LDL-C: low density lipoproteins-cholesterol
mg/kg: milligram per kilogram
mmol/l: milimol per liter
mRNA: messenger ribonucleic acid
NPD: normal pellet diet
OGTT: oral glucose tolerance test
qRT-PCR: quantitative real-time polymerase chain reaction
RNA: ribonucleic acid
SPSS: Statistical Package for the Social Sciences
STZ: streptozotocin
TC: total cholesterol
TG: triglycerides
